# Supplementary material for: LLPS REDIFINE allows the biophysical characterization of multicomponent condensates without tags or labels
Source: Nat Commun. 2025 May 19;16:4628. doi: 10.1038/s41467-025-59759-2 (PMC12089286; doi:10.1038/s41467-025-59759-2)
Supplement: Supplementary file 2 — Reporting Summary [file 41467_2025_59759_MOESM2_ESM.pdf]

nature portfolio

Corresponding author(s): Mihajlo Novakovic, Leonidas Emmanouilidis, Frederic Allain

Last updated by author(s): Apr 16, 2025

Reporting Summary

Nature Portfolio wishes to improve the reproducibility of the work that we publish. This form provides structure for consistency and transparency in reporting. For further information on Nature Portfolio policies, see our Editorial Policies and the Editorial Policy Checklist.

Statistics

For all statistical analyses, confirm that the following items are present in the figure legend, table legend, main text, or Methods section.

n/a	Confirmed	
☐	☒	The exact sample size (*n*) for each experimental group/condition, given as a discrete number and unit of measurement
☐	☒	A statement on whether measurements were taken from distinct samples or whether the same sample was measured repeatedly
☒	☐	The statistical test(s) used AND whether they are one- or two-sided<br>*Only common tests should be described solely by name; describe more complex techniques in the Methods section.*
☒	☐	A description of all covariates tested
☒	☐	A description of any assumptions or corrections, such as tests of normality and adjustment for multiple comparisons
☐	☒	A full description of the statistical parameters including central tendency (e.g. means) or other basic estimates (e.g. regression coefficient) AND variation (e.g. standard deviation) or associated estimates of uncertainty (e.g. confidence intervals)
☒	☐	For null hypothesis testing, the test statistic (e.g. *F*, *t*, *r*) with confidence intervals, effect sizes, degrees of freedom and *P* value noted<br>*Give P values as exact values whenever suitable.*
☒	☐	For Bayesian analysis, information on the choice of priors and Markov chain Monte Carlo settings
☒	☐	For hierarchical and complex designs, identification of the appropriate level for tests and full reporting of outcomes
☒	☐	Estimates of effect sizes (e.g. Cohen's *d*, Pearson's *r*), indicating how they were calculated

Our web collection on statistics for biologists contains articles on many of the points above.

Software and code

Policy information about availability of computer code

Data collection	Topspin 4.13 (Bruker), ND-1000 v3.8.1 (Thermo Fisher Scientific), Nikon Spinning Disk SoRa microscope
Data analysis	NMR data processing in Topspin 4.06 and analysis using custom-written code in Matlab version 2020b (https://doi.org/10.5281/zenodo.15228787). Microscopy images processing in ImageJ. Final figures are made in Illustrator 2024

For manuscripts utilizing custom algorithms or software that are central to the research but not yet described in published literature, software must be made available to editors and reviewers. We strongly encourage code deposition in a community repository (e.g. GitHub). See the Nature Portfolio guidelines for submitting code & software for further information.

Data

Policy information about availability of data

All manuscripts must include a data availability statement. This statement should provide the following information, where applicable:

- Accession codes, unique identifiers, or web links for publicly available datasets
- A description of any restrictions on data availability
- For clinical datasets or third party data, please ensure that the statement adheres to our policy

All raw NMR data presented in this work have been deposited to Zenodo repository [https://doi.org/10.5281/zenodo.15228787]. The Matlab codes used for simulations and fitting have been deposited to the same Zenodo repository [https://doi.org/10.5281/zenodo.15228787].

Research involving human participants, their data, or biological material

Policy information about studies with human participants or human data. See also policy information about sex, gender (identity/presentation), and sexual orientation and race, ethnicity and racism.

Reporting on sex and gender	/
Reporting on race, ethnicity, or other socially relevant groupings	/
Population characteristics	/
Recruitment	/
Ethics oversight	/

Note that full information on the approval of the study protocol must also be provided in the manuscript.

Field-specific reporting

Please select the one below that is the best fit for your research. If you are not sure, read the appropriate sections before making your selection.

☒ Life sciences ☐ Behavioural & social sciences ☐ Ecological, evolutionary & environmental sciences

For a reference copy of the document with all sections, see nature.com/documents/nr-reporting-summary-flat.pdf

Life sciences study design

All studies must disclose on these points even when the disclosure is negative.

Sample size	No statistical size calculation was performed due to the quantitative nature of the NMR method used. For turbidity, EMSA, microscopy experiments, we performed at least 3 independent measurements to comply with statistical significance.
Data exclusions	No data were excluded. All replications were succsessful.
Replication	Turbidity measurements - 3 biological replicates. EMSA assays - 3 technical replicates. Due to very reproducable nature of NMR measurements, most of the experiments were measured once. Repeatability of REDIFINE was probed with two NMR samples by measuring the experiment and performing independent fitting procedure three times. Specifically when biological replicability is tested, experiments were measured multiple times (>=3).
Randomization	No experimental groups in this study.
Blinding	Blinding was not used as all the data are objectively subjected to the code for automatized fitting. For microscopy, FRAP data and droplets coutning is also performed using a standard, authomatic procedure using same criteria.

Reporting for specific materials, systems and methods

We require information from authors about some types of materials, experimental systems and methods used in many studies. Here, indicate whether each material, system or method listed is relevant to your study. If you are not sure if a list item applies to your research, read the appropriate section before selecting a response.

Materials & experimental systems

n/a	Involved in the study	
☒	☐	Antibodies
☒	☐	Eukaryotic cell lines
☒	☐	Palaeontology and archaeology
☒	☐	Animals and other organisms
☒	☐	Clinical data
☒	☐	Dual use research of concern
☒	☐	Plants

Methods

n/a	Involved in the study	
☒	☐	ChIP-seq
☒	☐	Flow cytometry
☒	☐	MRI-based neuroimaging

Plants

Seed stocks

/

Novel plant genotypes

/

Authentication

/
